# Dynamics of genetic variability in *Anastrepha fraterculus *(Diptera: Tephritidae) during adaptation to laboratory rearing conditions

**DOI:** 10.1186/1471-2156-15-S2-S14

**Published:** 2014-12-01

**Authors:** María A Parreño, Alejandra C Scannapieco, María I Remis, Marianela Juri, María T Vera, Diego F Segura, Jorge L Cladera, Silvia B Lanzavecchia

**Affiliations:** 1 Laboratorio de Genética de Insectos de Importancia Económica, Instituto de Genética 'Ewald A. Favret', Instituto Nacional de Tecnología Agropecuaria, Hurlingham, Buenos Aires, Argentina; 2 Genética de la Estructura Poblacional, Departamento de Ecología, Genética y Evolución, Facultad de Ciencias Exactas y Naturales, Universidad de Buenos Aires; Buenos Aires, Argentina; 3 Cátedra Terapéutica Vegetal, Facultad de Agronomía y Zootecnia, Universidad Nacional de Tucumán, Tucumán, Argentina; 4 Consejo Nacional de Investigaciones Científicas y Técnicas, Ministerio de Ciencia, Tecnología e Innovación Productiva, Argentina

**Keywords:** fruit fly, adaptation, microsatellites, genetic variability, *A. fraterculus*, SIT

## Abstract

**Background:**

*Anastrepha fraterculus *is one of the most important fruit fly plagues in the American continent and only chemical control is applied in the field to diminish its population densities. A better understanding of the genetic variability during the introduction and adaptation of wild *A. fraterculus *populations to laboratory conditions is required for the development of stable and vigorous experimental colonies and mass-reared strains in support of successful Sterile Insect Technique (SIT) efforts.

**Methods:**

The present study aims to analyze the dynamics of changes in genetic variability during the first six generations under artificial rearing conditions in two populations: a) a wild population recently introduced to laboratory culture, named TW and, b) a long-established control line, named CL.

**Results:**

Results showed a declining tendency of genetic variability in TW. In CL, the relatively high values of genetic variability appear to be maintained across generations and could denote an intrinsic capacity to avoid the loss of genetic diversity in time.

**Discussion:**

The impact of evolutionary forces on this species during the adaptation process as well as the best approach to choose strategies to introduce experimental and mass-reared *A. fraterculus *strains for SIT programs are discussed.

## Introduction

The introduction of species into artificial conditions with the aim to establish laboratory lines is necessary and useful in numerous situations for experimental or mass rearing purposes. Laboratory conditions are different from the ones a species encounters in nature and often favor a small group of individuals with specific reproductive advantages [1]. For insect species, it has been observed that the adaptation to laboratory conditions frequently favors individuals with faster life cycles, females with high fecundity at the beginning of the reproductive stage, and males that do not necessarily accomplish all parts of the courting sequences /courtship [2-4]. At a molecular level, changes in the genetic variability have also been observed during the adaptation to laboratory conditions [5-11]. Specifically, the reduction of the population effective size in combination with the confinement to finite spaces has high impact on the genetic variability [12]. These changes could lead to a reduction in the ability of the species to confront changes in the environment or to survive if returned to wild conditions [13,14].

The adaptation of wild populations to laboratory conditions is a crucial step in modern biological control programs, such as in the case of the Sterile Insect Technique (SIT) [15-17]. The implementation of SIT requires the mass rearing and sterilization of insects for release into the natural environment. Once in the field, sterile males are expected to survive and mate with wild females. This leads to no offspring and therefore the population size is effectively reduced in time [18]. The quality control of the insects that are released is of vital importance to the success of the SIT. Therefore, it is important to assess the processes that may drive genetic changes during the introduction and adaptation of insects to laboratory conditions and the impact of these changes on life-history traits.

The South American fruit fly *Anastrepha fraterculus *Wiedemann (Diptera: Tephritidae), is among the targeted species of integral pest control programs in Latin America, given its great economic importance. Although SIT is currently being applied in Central America to control the population density of other species of *Anastrepha *genus [19-21], only chemical approaches have been used to control *A. fraterculus *populations [22]. This species infests a wide variety of fruit species, therefore being the reason of high crop losses and restrictions to potential markets, especially for Uruguay, Argentina, Peru and South of Brazil [23,24]. Currently, *A. fraterculus *is considered a complex of cryptic species (see [25-27]). In Argentina and southern Brazil, only one biological entity is described as *A. fraterculus *sp. 1 [26] or Brazilian 1 morphotype [27,28]. Numerous studies about biological, behavioral and genetic traits of this species have been carried out in the last 15 years as they are requirements before SIT can be applied to control this pest [29-32]. Viscarret *et al*. [33] were the first to assess the effects of adaptation to laboratory conditions in *A. fraterculus *sp. 1 populations. These authors found that recently introduced populations suffer drastic changes in survival and fecundity parameters.

Despite the limited information at genetic level available for this species, we have recently developed a set of species-specific microsatellite markers [34]. The development and availability of these markers are of major importance as very powerful tools for population genetics analyses [35,36]. In fact, these markers have been successfully used to assess intra-specific genetic diversity in wild and laboratory populations of this pest [34]. As a first approach to understand the dynamics of change of the genetic variability during the first generations under laboratory conditions in *A. fraterculus *sp. 1, we used a subset of these markers to analyze the genetic variability and differentiation of a laboratory and a wild population of this pest across six generations in the laboratory environment. We also discuss the driving evolutionary processes occurring during early laboratory adaptation and make suggestions regarding the maintenance of the natural vigor of laboratory populations aiming to support SIT development for *A. fraterculus*.

## Methods

### Introduction and adaptation of lines

Wild individuals were obtained in March 2012 from infested guava (*Psidium guajava *L.) in the vicinity of Tafi Viejo (Horco Molle, Tucumán, Argentina). The infested fruit was carried to the laboratory and placed in containers with sand as a substrate for pupation. Pupae were retrieved from the sand weekly and placed into glass containers. Emerged adults were introduced in 40-cm^3 ^cages, with water and food (MP Biomedical hydrolyzed yeast as protein source and sugar) to establish a replicated parental line named Tucumán Wild (TW). Each replicate population (TW1, TW2) was started with 760 individuals at a sex rate of 1:1. As control, a replicated parental line named Castelar Lab (CL) was started with pupae obtained from the experimental rearing at the Instituto de Genética 'Ewald A. Favret', Instituto Nacional de Tecnología Agropecuaria (IGEAF, INTA Castelar; Buenos Aires, Argentina). This laboratory strain was established in 2007 and maintained for 56 generations under artificial rearing according to Jaldo et al. [37], and since then no wild material has been introduced to refresh the genetic background. The strain was derived from a semi-mass rearing colony kept at Estación Experimental Agroindustrial Obispo Colombres, Tucumán, Argentina, originally initiated in 1997 with wild pupae recovered from infested guavas (*P. guajava *L.) collected at the vicinity of Tafi Viejo (Horco Molle, Tucumán, Argentina) [29].

Replication procedures for the CL populations (CL1, CL2) were similar to those described above for the TW line. TW and CL were kept isolated from each other for six generations under the same laboratory conditions (25°C, 45% humidity and 16-8 (L : D) photoperiod).

A cylindrical container (5 cm tall and 2 cm in diameter) filled with red colored water and covered by a plastic film was used as substrate for oviposition. These artificial fruits were checked every 24-48 h and the eggs were deposited into containers with artificial larval diet [38]. Containers were placed in other recipients containing sand as pupation substrate. Pupae were recovered after 17 days and placed in cages until adult emergence for the establishment of the next generation. Mean pupae-adult time was 15 days [37].

### Microsatellite genotyping methods

Replicates from each line, named as TW1, TW2, CL1 and CL2 respectively, were analyzed at generations 0, 3 and 6 (G_n_, G_n+3 _and G_n+6_) under laboratory rearing conditions. At each generation, 30-40 randomly chosen flies (in a 1:1 sex ratio) were obtained from each of the four populations to perform molecular analysis.

Individuals were kept at -20°C until processed. DNA was extracted following the protocol described by Baruffi et al. [39]. The genetic material was analyzed by electrophoresis (agarose 0.8 % in buffer TBE 0.5 × and revealed with ethidium bromide [40]). Images were captured with an UVP reveler (Fotodyne Inc. Hartland, WI, USA) and analyzed with Photoshop (Adobe Microsoft). The DNA was quantified with Nanodrop 1000 (Thermo Scientific).

The genetic variability of each population was analyzed with 10 microsatellite markers (A7, A120, A102, D12, A10, C103, D105, A122, A112 and D4) developed in Lanzavecchia et al. [34]. These microsatellite markers were selected from a set of 14 highly polymorphic microsatellites tested in wild and laboratory *A. fraterculus *populations [34]. Forward primers were 5´-labeled with fluorescent dyes (FAM-HEX). PCR reactions were done in a final volume of 10 µl containing: 2 mM dNTPs, 1.5 mM MgCl2, 0.4U *Taq *DNA polymerase (Inbio Highway, Tandil, Argentina) and 0.5 µM of each primer, using 40 ng of total DNA as template. Amplification was carried in a thermal cycler (MultiGene, Labnet, USA) with a denaturalization step of 2 minutes at 94°C followed by 29 cycles of 30 seconds at 94°C, 30 seconds at 58-60°C (see optimized annealing temperature [34]) and 30 seconds at 72°C, with a final elongation step of 10 minutes at 72°C. PCR products were evaluated with electrophoresis (agarose 1.5 % w/v) and run in an ABI 3130 XL DNA Analyzer (Applied Biosystems, Life Technologies, USA) with GeneScan 500 ROX Size Standard (Applied Biosystems). The results were processed using GeneMarker Software [41] to assign the genotype to each sample for each locus. All allele scores were visually inspected.

### Microsatellite data analysis

Genetic variability of each population at each generation was measured as: mean number of alleles per locus (Na), mean observed heterozygosity (Ho), expected heterozygosity (He) and allelic richness (AR) using FSTAT 2.9.3 [42] and Arlequin v3.11 [43].

Departures from Hardy-Weinberg equilibrium (HWE) for each locus and each population were quantified using the intra-population fixation index (F_IS_). The statistical significance of F_IS _values was assessed using the randomization procedure implemented in FSTAT [42] and afterward sequential Bonferroni corrections for multiple comparisons were applied [44]. The frequencies of null alleles for each locus and linkage disequilibrium between pairs of loci were estimated using Microchecker [45] and FSTAT [42].

To assess the initial genetic variation between the parental populations (TW and CL at G_n_) three different approaches were performed taking into account all *loci*: i) a hierarchical Analysis of Molecular Variance (AMOVA) using Arlequin [43]; ii) a genotypic differentiation test (Exact G test) implemented in GENEPOP 3.4 [46,47]; and iii) the Wilcoxon Matched Pairs Test run in STATISTICA [48].

The genetic diversity of the three generations analyzed in each population (CL and TW) was compared through Friedman non-parametric ANOVA using STATISTICA [48].

To quantify the distribution of total variation among generations within lines (G_n_, G_n+3 _and G_n+6_), among replicates within generations and within replicates, individual AMOVAs were performed for each line (TW and CL) with Arlequin [43].

Effects of positive selection were tested for each microsatellite locus by applying the Ln RH test [49,50]. This test detects loci with a pattern of variability which is different from that expected under neutrality, and is based on the comparison of the logarithm of the ratio between expected heterozygosities obtained for each locus in two populations: Ln RH = [((1/(1- H pop1))^2 ^- 1)/((1/(1- Hpop2))^2 ^- 1)]. To apply the test, ratios of expected heterozygosities were calculated for each locus using data from G_n _and G_n+6 _(G_n+6_/G_n _ratios) and for each line (CL and TW). Since Ln RH values are expected to follow a normal distribution for neutrality evolving microsatellite loci [49], significant deviations of Ln RH values from the Z distribution indicate positive selective sweep. To detect loci under selection, a one-way ANOVA was also performed with locus as a factor and Ln RH values as dependent variable according to Simões at al. [51]. These analyses were done using STATISTICA [48].

## Results

Over the four populations (TW1, TW2, CL1 and CL2 ), the ten microsatellite loci had different levels of polymorphism in terms of number of alleles, ranging from 2 to 15 and with a mean of 5.71 alleles per locus (data not shown). No linkage disequilibrium was detected with consistency across the populations (*P *> 0.05 in all cases). After the sequential Bonferroni correction, most of the populations sampled conformed to HWE at most of the loci. Out of the 120 tests performed (4 populations × 3 generations × 10 loci), only eight showed deviations from HWE (P < 0.05), measured using the fixation index (F_IS_) (Table S1, Additional file 1). The locus/population combinations that were not in HWE were not concentrated in any population or at any locus. Moreover, null alleles appeared to be present in various combinations of population/marker, which suggests that null alleles contribute to the heterozygote deficiency observed for some of these samples that exhibited deviations from HWE (Table S1, Additional file 1).

**Figure 1:**
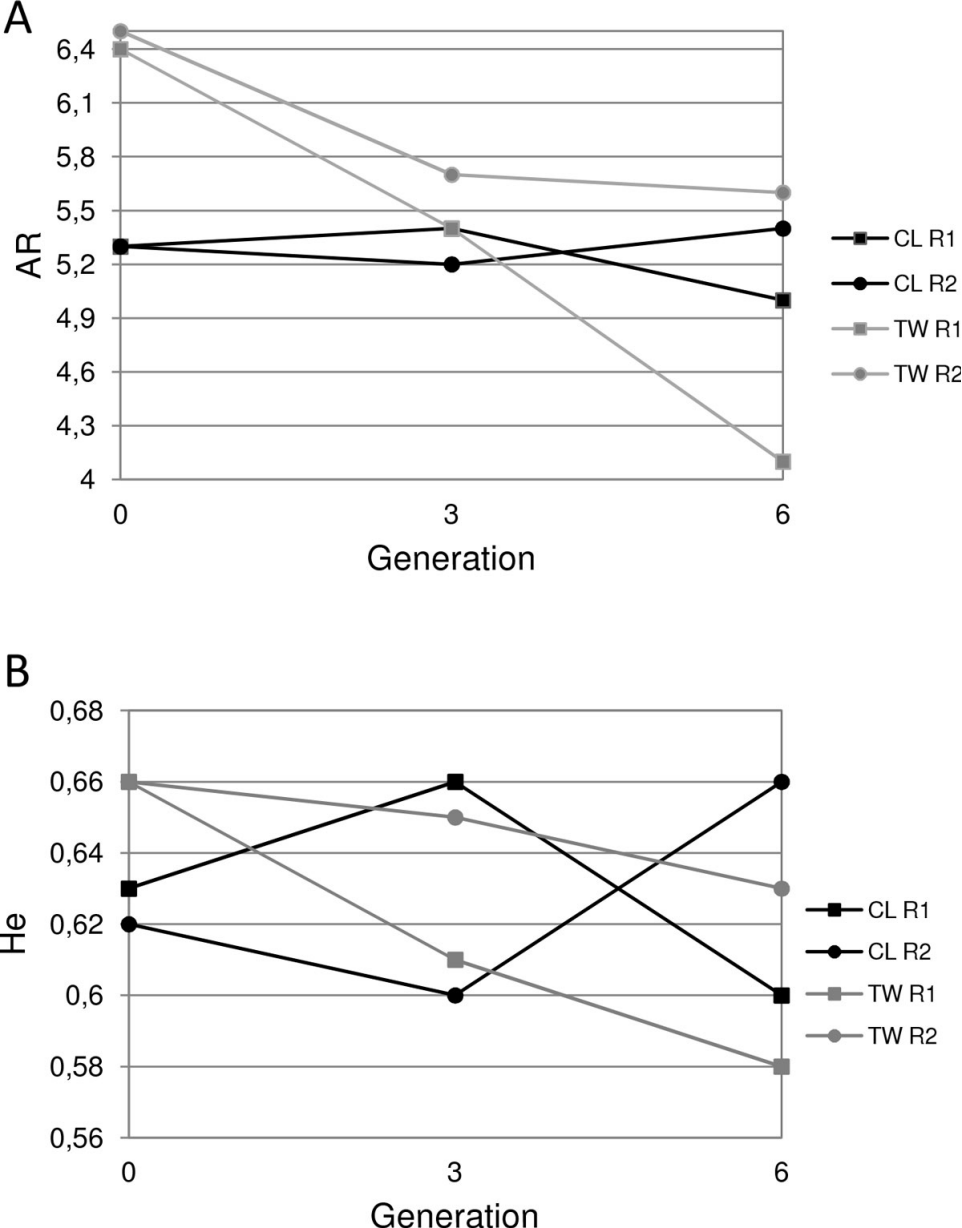
**Mean allelic richness, AR (A), and mean expected heterozygosity, He (B), at generations n, n+3 and n+6 under laboratory conditions of rearing for the four populations of *A. fraterculus***.

The two lines analyzed here, the long-established CL and the recently introduced TW, showed genetic heterogeneity. The AMOVA performed in G_n _detected significant differences between the lines (*F *= 0.0196; *P *= 0.00098), with 2% of genetic variability explained by this source of variation. The results from the Exact G test considering all loci in this generation also showed that genotypic frequencies differ between the lines (*P*<0.05). In addition, the genetic variability parameters showed high initial values for these lines. Particularly, TW seemed to show higher diversity in terms of mean number of alleles per locus (Na), allelic richness (AR) and expected heterozygosity (He) than CL in the parental generation G_n _(Table 1; Figure 1A,B), with marginally significant differences for AR between lines (Z = 1,72; *P *= 0.08, Wilcoxon Matched Pairs Test).

**Table 1:** Genetic variability estimates in the laboratory established line (CL) and the adapting line (TW) of *Anastrepha fraterculus *for generations n, n+3 and n+6 under laboratory rearing conditions.

Line and replicate	Generation	Na	AR	Ho	He
CL R1	G_n_	5.4 (2.4)	5.3	0.54 (0.28)	0.63 (0.17)
	G_n+3_	5.7 (2.4)	5.4	0.57 (0.18)	0.66 (0.15)
	G_n+6_	5.1 (2.4)	5.0	0.53 (0.26)	0.60 (0.22)
CL R2	G_n_	5.4 (2.7)	5.3	0.58 (0.28)	0.62 (0.23)
	G_n+3_	5.7 (2.3)	5.2	0.52 (0.20)	0.60 (0.18)
	G_n+6_	6.1 (2.6)	5.4	0.51 (0.15)	0.66 (0.13)
					
TW R1	G_n_	6.7 (2.9)	6.4	0.62 (0.12)	0.66 (0.16)
	G_n+3_	6.1 (2.3)	5.4	0.58 (0.20)	0.61 (0.18)
	G_n+6_	4.3 (1.9)	4.1	0.51 (0.26)	0.58 (0.18)
TW R2	G_n_	6.7 (2.8)	6.5	0.58 (0.24)	0.66 (0.18)
	G_n+3_	6.2 (2.9)	5.7	0.57 (0.16)	0.65 (0.14)
	G_n+6_	6.3 (2.8)	5.6	0.56 (0.25)	0.63 (0.21)

Na, mean number of alleles; AR, mean allelic richness; Ho, mean observed heterozygosity; He, mean expected heterozygosity. In parentheses: standard deviation.

The genetic diversity in CL seemed to be more stable through generations than that in TW, specifically in terms of AR (Table 1; Figure 1A). He for CL showed the same pattern as AR but with some inter-replicate variation (Figure 1B). In contrast, in TW, both variability parameters tended to decline during early laboratory adaptation (Figure 1A,B). Despite the high inter-replica variation detected within TW, marginally significant differences were detected in genetic variability across generations (Table 2). Particularly, one replicate of this line (TW R1) showed a stronger decline in genetic variability across generations than the other replicate (TW R2) (Figure 1A,B). The Friedman ANOVA demonstrated significant differences in both Na and AR among generations for the R1 replicate (Q = 6.35 *P *= 0.04 for Na; Q = 7.74, *P *= 0.02, for AR). Although these parameters showed a similar trend to decline through generations in R2, no significant differences were detected for this replicate. For CL, no significant differences were detected in genetic diversity indexes among generations (*P *> 0.05 in all cases).

**Table 2:** Results of the hierarchical analysis of molecular variance (AMOVA) for the CL and TW lines of *A. fraterculus*

Source of variation	Degree of freedom	% Total variation	Fixation indices	*P*
CL line				
Among generations	2	3.16	F_CT _= 0.03158	0.066
Among replicates Within generations	3	2.64	F_SC _= 0.02724	< 0.001
Within replicates	310	94.20	F_ST _= 0.05795	< 0.001
				
TW line				
Among generations	2	1.15	F_CT _= 0.0115	0.056
Among replicates Within generations	3	2.65	F_SC _= 0.0268	< 0.001
Within replicates	314	96.20	F_ST _= 0.1148	< 0.001

The degree of freedom, the percentage of variation, the *F *statistics and the probability (*P*) estimated from the permutation test are given at each hierarchical level.

AMOVA results (Table 2) support the pattern observed in the genetic diversity parameters analyzed, with low but marginally significant differences among generations. The differences between replicates within lines explained 2.65% and 2.64% of the total variance for TW and CL, respectively (*P*<0.05, Table 2). Most of the variation was found within replicates, with 96.20% (TW) and 94.20% (CL) of the total variance explained by this source (Table 2).

**Figure 2:**
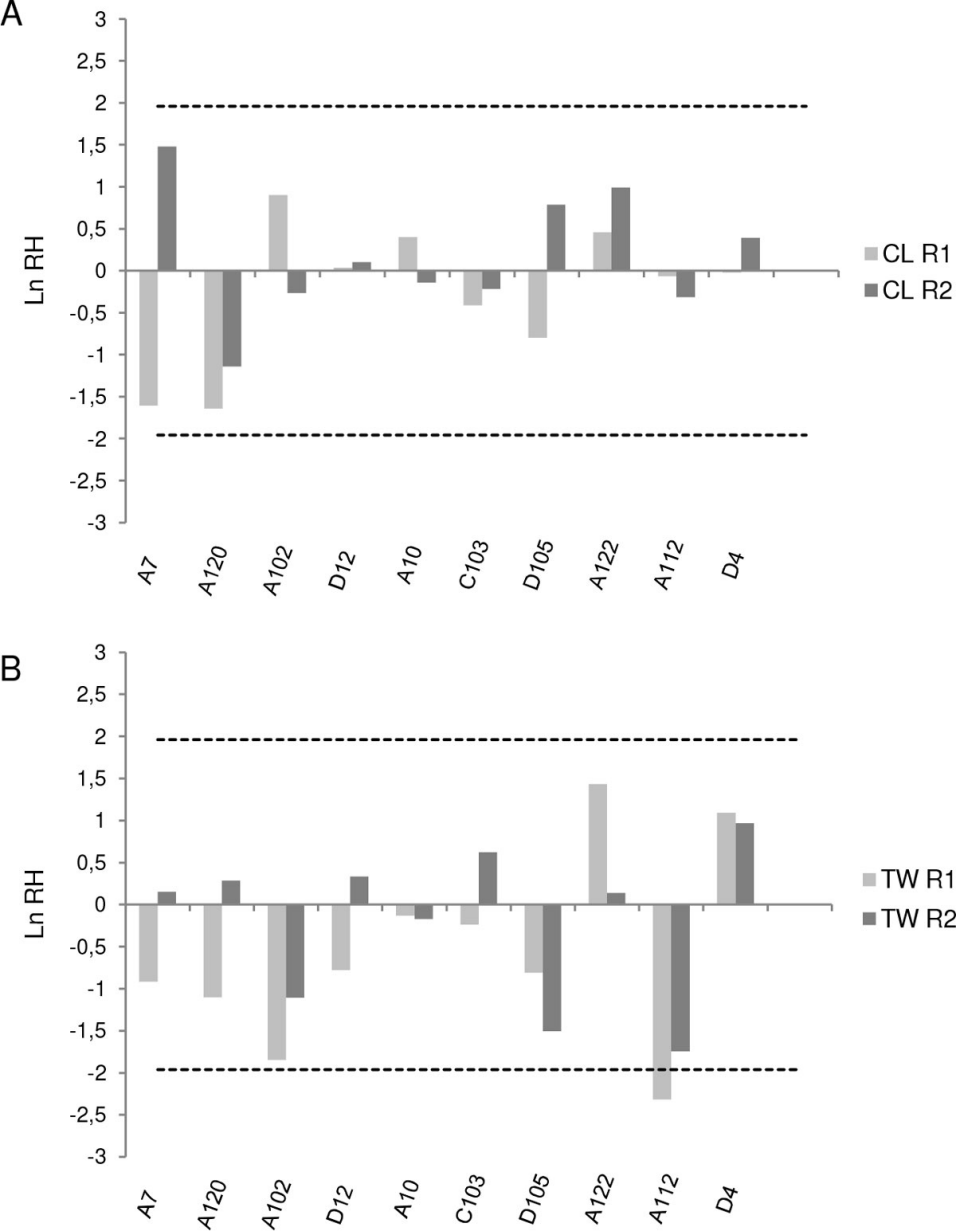
**Heterozygosity ratios (Ln RH) between generations n and n+6 for CL (A) and TW (B) populations**. Dashed lines represent the 95% confidence interval of the normal distribution. Positive and negative Ln RH values denote an increase or a decrease in variability through time, respectively.

Figure 2 shows the results obtained for the Ln RH test applied to detect deviations from neutrality in our microsatellite data set. No microsatellite locus exhibited any consistent indication of positive selection, except for locus A112 in R1 TW, whose Ln RH value fell slightly outside the 95% limits of the normal distribution. This locus showed a decrease in variability through generations in this TW replicate (Figure 2).

## Discussion

We presented here the first exploratory analysis of the dynamics of change in genetic variability for a wild *A. fraterculus *sp.1 population during early adaptation. We discuss our data in the frame of recent studies on other Tephritid species and explored the relative impact of evolutionary forces during laboratory adaptation. Our results indicate that during introduction to laboratory conditions *A. fraterculus *suffers a clear genetic differentiation, even in the very early steps of the adaptation process. Genetic drift appeared to be the main evolutionary force impacting on the genetic variability of the *A. fraterculus *during the introduction to laboratory rearing conditions.

The genotypic differences exhibited in the parental generation between CL and TW reflect the occurrence of genotypic variation between the long-established population and the wild population that gave origin to it. In line with previous studies, the wild population showed higher values in genetic diversity parameters. Our lab-adapted line also showed an overall high genetic variability in the 10 microsatellite loci, as described for laboratory strains of *Bactrocera dorsalis *Hendel [52], and a stable pattern of genetic variability across generations. These results evidence that the more than 56 generations in laboratory culture would have relatively low impact in the genetic diversity of this *A. fraterculus *experimental line. Moreover, the expected heterozygosity values were comparable between our CL line and the IGEAF established strain that gave origin (results obtained for 14 microsatellites markers) [35], suggesting the existence of an intrinsic mechanism that contributes to maintaining the variability in *A. fraterculus *even under laboratory conditions.

The declining patterns in genetic variability through early adaptation to laboratory conditions observed for TW are consistent with the results obtained by Gilchrist et al. [10] and Zigouridis et al. [11] in *Bactrocera *spp. In larger-scale studies, these authors followed the fluctuation of genotypic frequencies of microsatellite markers during the colonization of replicated lines of *Bactrocera tryoni *Froggat (Diptera: Tephritidae) and a wild population of *B. oleae *Rossi (Diptera: Tephritidae) in laboratory conditions, respectively. Substantial changes in the genetic variability during the initial period of adaptation were observed for these species, particularly between generations 4 and 10 for *B. tryoni *[10] and generations 1 and 5 for *B. oleae *[11].

The high inter-replicate differences observed particularly for our TW line suggest the occurrence of genetic drift with detectable impact on the genetic variability of the replicates, as described by Gilchrist et al. [10] for *B. tryoni *and Simões et al. [8,51,53] for *Drosophila subobscura *Collin (Diptera: Drosophilidae) populations. In agreement with the study by Hopper with small population sizes [54], genetic drift appears to be the main evolutionary process involved in the genetic differentiation of our TW populations.

We found no strong evidence of selection with the Ln RH test applied to the microsatellite data. Only one locus (A112) showed slight deviations from neutral expectations at one TW replicate (R1) between Gn and Gn+6. Our results point out the relative importance of genetic drift, particularly, through the smaller effective population sizes at early stages of adaptation, molding genetic molecular diversity in our recently introduced TW line, although certain genome region (A112 locus) could reflect selection during laboratory adaptation. In this context, we suggest that the implementation of strategies like the cross of replicates, as proposed by Gilchrist et al. [10,55], could maintain variability in regular *A. fraterculus *experimental and mass-rearing programs. The approach proposed by these authors relates to the fragmentation of a population to diminish the genetic adaptation to captivity [7], which may reduce the genetic variability at a replicate level but maintain it at a population level. Keeping isolated replicates and pooling them every certain number of generations would help to reduce inbreeding to an acceptable level [56]. Additionally, results presented by Zygouridis et al. [11] indicate that refreshments with wild material performed every five to eight generations could be adequate to maintain a wild profile in terms of genetic variability in a *B. oleae *mass-reared colony and would represent a good strategy to maintain variability in this species. In the case of our *A. fraterculus *line, the adequate number of generations between replicate crosses or refreshments with wild material and the evolutionary forces driving genetic changes in the adaptation process of *A. fraterculus *deserve further evaluation including a larger number of generations and a larger number of replicates. The possible combination of these studies with other management strategies for *A. fraterculus *remains as a challenge for future research and will provide useful information to establish improved and efficient experimental and mass-rearing colonies for the development of SIT for this pest.

The present study represents a first insight into the dynamics of change in the genetic variability of *A. fraterculus *populations during adaptation to laboratory conditions. Our results indicate the relative importance of genetic drift as a principal driving evolutionary force in early laboratory adaptation that must be further analyzed. Given our findings of the dissimilarities between replicates, we suggest that the fragmentation and crosses among replicates after several generations may be a suitable procedure to maintain the genetic diversity of the population in captivity. Future challenges should include analyzing further generations and establishing the association between long-term dynamics of change in the genetic variability and in biological or behavioral parameters of relevance to the quality assessment of mass-reared colonies (as fecundity, mating performance, etc) across generations for SIT implementation against this pest.

## Conclusion

Studies about changes in genetic variability during adaptation to artificial rearing are of fundamental importance to the monitoring of biological and genetic parameters applicable to mass-reared strains in the frame of SIT control strategies. These studies bring useful information about genetic aspects of foundation and management strategies for *A. fraterculus *experimental rearing. We introduced here a first approach to the genetic changes of a wild population of *A. fraterculus *during the first generations under artificial rearing. Based on recent studies on other Tephritidae species, we discuss the possible evolutionary forces driving the genetic changes observed here. Although our investigations are based on an experimentally reared strain, the results provide useful information about the genetic aspects of this species that could be useful in the development of larger-scale rearing to maximize its potential in the field when specific control strategies are applied.

As SIT has not yet been developed for *A. fraterculus*, all the investigations focused on this direction, as are biological and genetic studies performed in natural and lab strains, will be of paramount importance to help governmental decisions to solve the important economic problem posed by this fruit pest in South America.

## Competing interests

The authors declare that they have no competing interests.

## Authors' contributions

MAP performed the experimental procedures, molecular genetic studies and participated in the drafting of the manuscript. ACS performed the genetic and statistical analysis and drafted the manuscript. MIR performed the statistical analysis and helped to draft the manuscript. MJ participated in the characterization of the microsatellite markers and molecular genetic studies. MTV and DFS participated in the experimental design and coordinated the management of insect strains. JLC participated in the design of the study and helped to draft the manuscript. SBL participated in the design of the study, genetic analysis and drafting of the manuscript. All authors read and approved the final version of the manuscript.

## Supplementary Material

Additional file 1 Table S1 (Additional_file1_Table1_Parreño et al. 2014.xls). Table S1. Fixation index F_IS _and null allele frequency at each locus for all populations (CL R1, CL R2, TW R1, TW R2) and generations (n, n+3, n+6). Bold values represent significant frequencies of null alleles after Bonferroni correction (P < 0.05). F_IS _values with asterisk represent significant deviations from HWE, *P < 0.05. Click here for file

## References

[B1] Lacy RC Clarification of genetic terms and their use in the management of captive populations Zoo Biol 1995 14 565 578 10.1002/zoo.1430140609

[B2] Carey JR Host-specific demographic studies of the Mediterranean fruit fly *Ceratitis capitata* Ecol Entomol 1984 9 261 270 10.1111/j.1365-2311.1984.tb00850.x

[B3] Joachim-Bravo IS Zucoloto FS Performance and feeding behavior of *Ceratitis capitata*: comparison of a wild population and laboratory population Entomol Exp Appl 1998 87 67 72 10.1046/j.1570-7458.1998.00305.x

[B4] Economopoulos AP Adaptation of the Mediterranean Fruit Fly (Diptera: Tephritidae) to Artificial Rearing J Econ Entomol 1992 85 Suppl 3 753 758

[B5] Dupont-Nivet M Mallard J Bonnet J Blanc J Evolution of the genetic variability in a population of the edible snail, *Helix aspersa *Muller, undergoing domestication and short-term selection Heredity 2001 87 129 135 10.1046/j.1365-2540.2001.00836.x 11703502

[B6] Ekesi S Nderitu PW Chang CL Adaptation to and Small-Scale Rearing of Invasive Fruit Fly *Bactrocera invadens *(Diptera: Tephritidae) on Artificial Diet Ann Entomol Soc Am 2007 100 Suppl 4 562 567

[B7] Frankham R Genetic adaptation to captivity in species conservation programs Mol Ecol 2008 17 325 333 10.1111/j.1365-294X.2007.03399.x 18173504

[B8] Simões P Pascual M Coelho M Matos M Divergent evolution of molecular markers during laboratory adaptation in *Drosophila subobscura* Genetica 2010 138 999 1009 10.1007/s10709-010-9486-4 20803349

[B9] Goncalves EC Ferrari SF Bastos HB Wajntal A Aleixo A Schneider MP Comparative genetic diversity of wild and captive populations of the bare-faced curassow (*Craxfa sciolata*) based on cross-species microsatellite markers: implications for conservation and management Biochem Genet 2010 48 Suppl 5-6 472 479 20087657 10.1007/s10528-010-9330-7

[B10] Gilchrist AS Cameron EC Sved JA Meats AW Genetic consequences of domestication and mass rearing of pest fruit fly *Bactrocera tryoni *(Diptera: Tephritidae) J Econ Entomol 2012 105 Suppl 3 1051 1056 22812147 10.1603/ec11421

[B11] Zygouridis NE Argov Y Nemny-Lavy E Augustinos AA Nestel D Mathiopoulos KD Genetic changes during laboratory domestication of an olive fly SIT strain J Appl Entomol 2014 138 Suppl 6 423 432 Published online: 27 FEB 2013

[B12] Nei M Maruyama T Chakraborty R The bottleneck effect and genetic variability in populations Evolution 1975 29 1 10 10.2307/2407137 28563291

[B13] Bartlett AC King EG and Leppla NC Genetic changes during insect domestication Advances and Challenges in Insect Rearing 1984 New Orleans 2 8

[B14] Hernandez ED Toledo J Artiaga-Lopez T Flores S Demographic changes in *Anastrepha oblique *(Diptera: Tephritidae) throughout the laboratory colonization process J Econ Entomol 2009 102 Suppl 2 542 551 19449633 10.1603/029.102.0211

[B15] Knipling EF The basic principles of insect population suppression and management 1979 Agriculture Handbook Number 512, SEA, USDA, Washington DC, USA

[B16] Enkerlin WR Dyck VA, Hendrichs J, Robinson AS, Dordrecht Impact of fruit fly control programmes using the Sterile Insect Technique Principles and Practice in Area-Wide Integrated Pest Management 2005 The Netherlands: Springer 651 676

[B17] Hendrichs MA Wornoayporn V Katsoyannos B Hendrichs J Quality control methods to measure predator evasion in wild and mass-reared Mediterranean fruit flies (Diptera: Tephritidae) Fla Entomol 2007 90 Suppl 1 64 70

[B18] Aluja MR Bionomics and management of *Anastrepha* Annu Rev Entomol 1994 39 155 178 10.1146/annurev.en.39.010194.001103

[B19] Hernandez E Orozco D Breceda SF Dominguez J Dispersal and longevity of wild and mass reared *Anastrepha ludens *and *Anastrepha oblique *(Diptera: Tephritidae) Fla Entomol 2007 90 123 135 10.1653/0015-4040(2007)90[123:DALOWA]2.0.CO;2

[B20] Zepeda-Cisneros C Meza Hernández J Ibañez Palacios J García Martínez V León Crisóstomo A Development and evaluation of genetic sexing strain of *Anastrepha ludens *for sterile insect technique Report of the International Congress of Entomology: 20-24 August 2012 Daegu, Korea

[B21] Guillen D Sanchez R Vreysen M.B, Robinson AS, Hendrichs J Expansion of the national fruit control programme in Argentina Area-wide control of insect pests from research to field implementation 2007 IAEA 653 660

[B22] Aruani R Ceresa A Granados J Taret G Peruzzoti P Ortiz G McPheron B, Steck G Advances in the national fruit fly control and eradication program in Argentine Fruit Fly Pests: A World Assessment of their Biology and Management 1996 521 530

[B23] Sugayama R Kovaleski A Liedo P Malavasi A Colonization of a new fruit crop by *Anastrepha fraterculus *(Diptera: Tephritidae) in Brazil: a demographic analysis Environ Entomol 1998 27 642 648

[B24] Cosenzo E Programa nacional de control y erradicación de mosca de los frutos (PROCEM) Resúmenes del Taller de Mosca de los Frutos 2003 2003 Buenos Aires. SENASA

[B25] Steck G Taxonomic status of *Anastrepha fraterculus* Proceedings of the Workshop on the South American fruit fly, Anastrepha fraterculus (Wied.); advances in artificial rearing, taxonomic status and biological studies: 1-2 Nov 1996 1999 Viña del Mar, Chile 13 20

[B26] Goday C Selivon D Perondini ALP Greciano PG Ruiz MF Cytological characterization of sex chromosomes and ribosomal DNA location in *Anastrepha *species (Diptera, Tephritidae) Cytogenet Genome Res 2006 114 70 76 10.1159/000091931 16717453

[B27] Hernández-Ortiz V Bartolucci A Morales-Valles P Frías D Selivon D Cryptic Species of the *Anastrepha fraterculus *Complex (Diptera: Tephritidae): A Multivariate Approach for the Recognition of South American Morphotypes Ann Entomol Soc Am 2012 105 Suppl 2 135 376

[B28] Rull J Abraham S Kovaleski A Segura DF Islam A Wornoayporn V Dammalage T Tomas US Vera MT Random mating and reproductive compatibility among Argentinean and southern Brazilian populations of *Anastrepha fraterculus *(Diptera: Tephritidae) Bull Entomol Res 2012 102 435 443 10.1017/S0007485312000016 22360877

[B29] Jaldo H Gramajo C Willink E Mass rearing of *A. fraterculus*: A preliminary Strategy Fla Entomol 2001 84 716 718 10.2307/3496407

[B30] Vera MT Wood RJ Cladera J Gilburn AS Factors affecting female remating frequency in the Mediterranean fruit fly (Diptera: Tephritidae) Fla Entomol 2002 85 156 164 10.1653/0015-4040(2002)085[0156:FAFRFI]2.0.CO;2

[B31] Gomez Cendra P Segura D Allinghi A Cladera J Vilardi J Comparison of longevity between a laboratory strain and a natural population of *Anastrepha fraterculus *(Diptera: Tephritidae) under field cage conditions Fla Entomol 2007 90 147 153 10.1653/0015-4040(2007)90[147:COLBAL]2.0.CO;2

[B32] Vera MT Abraham S Oviedo A Willink E Demographic and quality control parameters of *Anastrepha fraterculus *(Diptera: Tephritidae) maintained under artificial rearing Fla Entomol 2007 90 53 57 10.1653/0015-4040(2007)90[53:DAQCPO]2.0.CO;2

[B33] Viscarret M Carabajal-Paladino LZ Lanzavecchia SB Milla FH Cladera JL Evaluación de parámetros biológicos de Anastrepha fraterculus (Diptera: Tephritidae) durante el proceso de adaptación a la cría artificial Proceedings of the VII Congreso Argentino de Entomología: 21-24 Oct 2008 Córdoba, Argentina

[B34] Lanzavecchia SBL Juri M Bonomi A Gomulski L Scannapieco AC Segura DF Malacrida A Cladera J Gasperi G Microsatellite markers from the 'South American fruit fly' *Anastrepha fraterculus*: a valuable tool for population genetic analysis and SIT applications BMC Genetic 2014 15 (Suppl 2) S13 10.1186/1471-2156-15-S2-S13 PMC4255783 25471285

[B35] Bruford MW Wayne RK Microsatellites and their application to population genetic studies Curr Opin Genet Dev 1993 3 939 943 10.1016/0959-437X(93)90017-J 8118220

[B36] Schlötterer C Pemberton J The use of microsatellites for genetic analysis of natural populations EXS 1994 69 203 214 7994107 10.1007/978-3-0348-7527-1_11

[B37] Jaldo HE Willink E Liedo P Demographic analysis of mass-reared *Anastrepha fraterculus *(Diptera: Tephritidae) in Tucumán, Argentina Revista Industrial y Agrícola de Tucumán 2007 84 15 20

[B38] Salles L Rearing of *Anastrepha fraterculus *(Wiedemann). In The South American fruit fly, *Anastrepha fraterculus *(Wied.); advances in artificial rearing, taxonomic status and biological studies Proceedings of a workshop organized by the Joint FAO/IAEA Division of Nuclear Techniques in Food and Agronomy: 1-2 November 1996 Vina del Mar, Chile

[B39] Baruffi L Damiani G Guglielmino C Bandi C Malacrida A Gasperi G Polymorphism within and between populations of *Ceratitis capitata*: comparison between RAPD and multi *locus *enzyme electrophoresis data Heredity 1995 74 425 437 10.1038/hdy.1995.60 7759289

[B40] Sambrook J Fritschi EF Maniatis T Molecular cloning: a laboratory manual 1989 2 New York: Cold Spring Harbor Laboratory Press

[B41] SoftGenetics LLC, GeneMarker version 2.4.0 http://www.softgenetics.com/GeneMarker.html

[B42] Goudet J FSTAT (Version 1.2): A Computer Program to Calculate F-Statistics J Hered 1995 86 Suppl 6 485 486

[B43] Excoffier L Laval G Scheneider S Arlequin (version 3.0): An integrated software package for population genetics data analysis Evol Bioinfor 2005 1 47 50 PMC2658868 19325852

[B44] Rice W Analyzing Tables of Statistical Tests Evolution 1989 43 Suppl 1 223 225 10.1111/j.1558-5646.1989.tb04220.x 28568501

[B45] Van Oosterhout C Hutchinson W Wills P Shirpley P Program Note Micro Checker: Software for identifying and correcting genotyping errors in microsatellite data Mol Ecol 2004 4 535 538 10.1111/j.1471-8286.2004.00684.x

[B46] Raymond M Rousset F GENEPOP (version 1.2): population genetics software for exact tests and ecumenicism Heredity 1995 86 248 249

[B47] Rousset F Genepop'007: a complete reimplementation of the Genepop software for Windows and Linux Mol Ecol Resources 2008 8 103 106 10.1111/j.1471-8286.2007.01931.x 21585727

[B48] Statistica v 5.1 Statsoft, Tulsa, OK, USA 1996

[B49] Schlötterer C A microsatellite-based multilocus screen for the identification of local selective sweeps Genetics 2002 160 753 763 11861576 10.1093/genetics/160.2.753 PMC1461965

[B50] Kauer MO Dieringer D Schlötterer C A microsatellite variability screen for positive selection associated with the 'Out of Africa' habitat expansion of *Drosophila melanogaster* Genetics 2008 165 1137 1148 14668371 10.1093/genetics/165.3.1137 PMC1462820

[B51] Simões P Pascual M Santos J Fragata I Rose M Matos M Evolutionay dynamics of molecular markers during local adaptation: a case study in *Drosophila subobscura* BMC Evolutionary Biology 2008 8 66 10.1186/1471-2148-8-66 18302790 PMC2266711

[B52] Aketarawong N Chinvinijkul S Orankanok W Guglielmino C Franz G Malacrida A Thanaphum S The utility of microsatellite DNA markers for the evaluation of area-wide integrated pest management using SIT for the fruit fly, *Bactrocera dorsalis *(Hendel), control programs in Thailand Genetica 2011 139 Suppl 1 129 40 21052785 10.1007/s10709-010-9510-8

[B53] Simões P Santos J Fragata I Mueller L Rose M Matos M How repeatable is adaptive evolution? The role of geographical origin and founder effects in laboratory adaptation Evolution 2008 62 1817 1829 10.1111/j.1558-5646.2008.00423.x 18489721

[B54] Hopper K Roush R Powell W Management of genetics of biological-control introductions Annu Rev Entomol 1993 38 27 51 10.1146/annurev.en.38.010193.000331

[B55] Gilchrist A Meats A The genetic structure of populations of an invading pest fruit fly, *Bactrocera tryoni*, at the species climatic range limit Heredity 2010 105 165 172 10.1038/hdy.2009.163 19997126

[B56] Frankham R Ballou J Briscoe D Introduction to conservation genetics 2002 Cambridge. UK: Cambridge University Press

